# Harnessing operating room signals to estimate mean arterial pressure with AnesthNet

**DOI:** 10.1038/s41598-025-12341-8

**Published:** 2025-09-30

**Authors:** Jade Perdereau, Jona Joachim, Fabrice Vallée, Jérôme Cartailler, Thomas Moreau

**Affiliations:** 1 INSERM, U942 MASCOT, Université Paris Cité, Paris, 75006 France; 2 Department of Anaesthesia and Critical Care, APHP, Hôpital Lariboisière, Paris, 75010 France; 3 Inria, Université Paris-Saclay, Palaiseau, France; 4 LMS, CNRS, Institut Polytechnique de Paris, Palaiseau, France

**Keywords:** Biomarkers, Biomedical engineering, Computer science

## Abstract

Monitoring mean arterial pressure (MAP) is essential for ensuring safe general anesthesia. Current practices rely either on non-invasive cuff measurements, which suffer from poor temporal resolution, or invasive arterial lines, which provide excellent accuracy and resolution but carry a significant risk of complications. Therefore, identifying alternatives to arterial lines in the operating rooms is a pressing need. Despite the importance of this issue in the community, clinically viable non-invasive MAP monitoring methods have yet to emerge. Existing approaches often encounter reproducibility issues, notably on large, open-source databases, and are not always optimized for real-time predictions. To address these limitations, this study introduces AnesthNet, a deep learning architecture designed for MAP estimation, using data exclusively from non-invasive and routine sensors such as photoplethysmography, ECG, and cuff oscillometer. AnesthNet was evaluated against the best-performing state-of-the-art deep learning architectures, using international standards to assess their performance on two of the largest datasets to date: VitalDB (2,833 patients) and LaribDB (5,060 patients). AnesthNet achieved superior performances, reaching an MAE of 4.6 (± 4.7) mmHg on VitalDB and 3.8 (± 5.7) mmHg on LaribDB. Our model also outperformed other architectures for different delays in cuff values and yielded no significant latency during inference, meeting clinical real-time requirements.

## Introduction

Maintaining appropriate blood pressure is essential to ensure adequate oxygen delivery to tissues. During general anesthesia, anesthetic drugs often induce a drop in blood pressure, potentially leading to intra-operative hypotension. Even brief episodes of hypotension are associated with serious adverse outcomes and economic strain, including longer hospital stays^1^, acute kidney injury, cardiac events^2,3^, and increased mortality^4^. Yet, arterial pressure monitoring is based on a compromise between the use of invasive sensors and the availability of continuous data to guide anesthetic and hemodynamic interventions^5,6^. Invasive sensors provide continuous and highly accurate measurements but are associated with a risk of complications and are often limited by their complexity, cost, and availability. Conversely, non-invasive cuff-based oscillometry is widely accessible but provides intermittent readings every 2 to 5 minutes, potentially missing transient yet clinically significant drops in MAP. Advancing arterial pressure monitoring requires developing methods that accurately track pressure changes between cuff measurements without invasive procedures.

Previous studies have demonstrated the potential of non-invasive vital signs measurements to estimate continuous blood pressure variations, primarily aiming to enable remote monitoring aid for hypertensive event detection. Notably, blood oxygenation measured at the fingertip through photoplethysmography (PPG) has been associated with blood pressure^7,8^ and used to estimate it continuously^9^. PPG-derived markers were shown to offer valuable insights for estimating continuous MAP^10^. However, the generalizability of such models on external and largest cohorts remains challenging. Despite these limitations, PPG remains a promising candidate for blood pressure estimation due to its well-established physiological basis. For instance, hand-crafted indicators derived from PPG waveform are characteristic of vascular resistance or tissue perfusion^11^. Other studies considered predicting the BP from the central measure of the electrocardiogram (ECG). However, ECG alone was not deemed a viable standalone option for blood pressure estimation^12^ and should be used in conjunction with other modalities. If Systolic Blood Pressure and Diastolic Blood Pressure are commonly overseen in outpatients, the standard metric used by anesthetists for blood pressure monitoring recommendations, with the international consensus recommendation to maintain MAP > 60 mmHg for high-risk patients^13^. Moreover, owing to MAP linear definition from systolic and diastolic pressures, and recent findings showing they share redundant information content under intraoperative conditions^1^, the scope of this study will be focused on MAP estimation. As for calibration, brachial cuff measurements are part of the operating room’s routinely acquired vitals. Considering the drifts observed for cuff-less blood pressure predictions^14^, operating-room applications strongly benefit from leveraging all available data, notably calibration using cuff oscillometry.

Reconstructing blood pressure from other vital signs requires capturing their nonlinear relationships. Many methodologies have been explored, including features-to-signal and signal-to-signal study designs. Features-to-signal methods are based on hand-crafted time-domain and frequency-domain features, measured or calculated on the waveform signal. They reduce the problem complexity by extracting key features from the vitals time series to predict blood pressure. They have mostly been developed with small-scale datasets (n < 100) using classical signal processing and machine learning techniques, such as spectral analysis^15^, wavelet transforms^16^, or random forest regression^17,18^. With the emergence of larger datasets, with thousands of records, deep learning-based methods have become increasingly popular, with various architectures proposed to tackle the signal-to-signal classification, based on class of neural networks such as multi-layer perceptron^19,20^, convolutional neural networks (CNN)^21,22^, recurrent networks such as long short term memory (LSTM) and GRU^23^, up to U-shaped architectures such as Wave-U-Net^24^. These methods leverage the increased sample sizes to learn models that better generalize.

Among these neural networks, the U-Net-based architectures have proven to provide the best results on the largest datasets at the time^25^, notably for signal-to-signal approaches, while combining CNN and LSTM layers was also found conclusive on a dataset of mixed inputs of PPG and ECG features^20^.

The latest advances in data collection infrastructures in the operating room have boosted the emergence of deep-learning-based models. Improved data collection led to the constitution and publication of databases composed of systematically recorded operating-room signals. The Multiparameter Intelligent Monitoring in Intensive Care (MIMIC) III dataset, composed of thousands of critical care unit (ICU) patients records of the Beth Israel Deaconess Medical Center, in Boston, Massachussetts, US, was first released in 2016^26^. More recently and specifically to anesthesia, VitalDB gathers 6,388 ICU patients who underwent surgeries in the Seoul National University Hospital, in South Korea^27^. The publication of such large datasets has been instrumental in validating international standards of the field, such as the Association for the Advancement of Medical Instrumentation (AAMI) standard defining validation datasets criteria with a minimal sample size of 85^28^.

While previous studies have explored arterial pressure estimation using physiological data, many have overlooked critical operating room constraints. Two fundamental challenges persist: maintaining causality and incorporating calibration effectively. First, causality must be preserved—a requirement violated by architectures like bidirectional LSTMs^8,29^. Although causal convolutional neural networks (e.g., TCNs)^30,31^ have been employed, they often lack long-term memory and fail to integrate physiological priors such as calibration. Second, while cuffless architectures have gained traction, their performance degrades significantly on large datasets or during hemodynamic fluctuations. Unlike wearable applications, where calibration is impractical, anesthesiologists require precise estimates leveraging all available intraoperative data. However, prior attempts to incorporate calibration often compromised causality. For instance, PPG2BP-Net^32^ uses random segment selection and future data access, rendering it unsuitable for real-world clinical use. Moreover, at inference time, the arterial line might be unavailable, questioning the deployability of such a solution. Additionally, methodological inconsistencies across studies, such as comparisons on disparate datasets, hinder objective performance evaluation.

To address these limitations, this study introduces AnesthNet, a deep learning architecture designed for real-time continuous MAP estimation in operating room settings. The key contributions of this work include: (1) the use of dilated causal CNNs to expand the receptive field and capture long-range dependencies while mitigating short-term memory limitations, (2) the integration of cuff-derived MAP values as initialization parameters to incorporate clinical knowledge into the model, and (3) rigorous benchmarking on standardized datasets including the open-source VitalDB dataset and our in-house LaribDB dataset, comprising over 10,000 intraoperative recordings. This custom dataset notably contains valuable physiological features such as the brachial cuff oscillometry or the perfusion index, which are missing from open source datasets. Performance was assessed using established international standards from the British Hypertension Society and the Association for the Advancement of Medical Instrumentation (AAMI).

To reflect the constraints of clinical application, the inference time of the proposed model was also assessed to ensure it can provide valuable real-time feedback, as well as the impact of distance to the cuff calibration on the performances.

## Methods

### Approval for data acquisition

The VitalDB dataset was downloaded using the provided Python library^27^. Data derived from the MIMIC-III matched subset are released under the Open Database License (ODbL), while data derived from the VitalDB dataset are released under the Creative Commons Attribution-NonCommercial-ShareAlike 4.0 International (CC BY-NC-SA 4.0) license.

As for LaribDB, it is a retrospective cohort study, conducted using the Assistance Publique-Hopitaux de Paris (AP-HP) Health Data Warehouse (“Entrepôt de Données de Santé (EDS),” https://eds.aphp.fr/). This data warehouse contains electronic health records (EHRs) of all inpatients from the 39 greater Paris University hospitals. The Institutional Review Board of the AP-HP Health Data Warehouse approved this study on April 2nd, 2024, under the number CSE-24-05_PLETHOMAP. This cohort included 10117 patient records in the Department of Anesthesiology and Critical Care at Lariboisière University Hospital from January 2018 to September 2023, stored at the Assistance Publique-Hopitaux de Paris (AP-HP) Health Data Warehouse deployed at the Lariboisière Hospital. Patient biometric data was monitored using a Philips IntelliVue MX850 bedside monitor (Philips, Eindhoven, The Netherlands), which was connected to the Data Warehouse Connect storage solution (Philips, Eindhoven, The Netherlands). This allowed for real-time, automated database storage of the data from the patient monitor for offline analysis. Data was recorded at a sampling rate of 1 Hz for low-frequency data and 125 Hz for high-frequency waveform data.

### Data selection/Pre-processing

#### Datasets generation

The patients relevant to this study from the VitalDB database were selected based on the simultaneous presence of PPG, ECG, HR and ABP signals. The records were generated using the Python vitaldb library and relevant features such as the notch relative amplitude and cycle MAP were calculated from ABP using the open-source physiocurve library. After the preprocessing, data was saved as patient-level PyTorch tensors to maximize GPU usage and enable parallel processing using the joblib library.

LaribDB was retrieved from the Data Warehouse Connect using the dwclib Python library, and its initial format was Parquet. Considering the high variability of patients’ records length, notably for intensive care data, some patient records reached over 100 gigabytes of data and would not fit in memory. To address such memory issues, Dask was used for chunk data reading, processing, and rewriting. The use of chunk data read resulted in a reduction of peak memory of 68% and an increase of memory efficiency - defined as the ratio of the actual data size to the peak memory usage - of 170%. Abnormal records were defined as records for which either of the features was unavailable, and the minimal patient record length was set to one hour.

#### Derived features generation and missing values

Derived features were generated from raw waveform data. The dicrotic notch calculated on the PPG waveform was generated using the open-source Python library physiocurve (https://framagit.org/jaj/physiocurve). These features The VitalDB dataset lacked certain features, specifically the perfusion index and brachial cuff measurements. To retrospectively generate the missing cuff values, LaribDB was utilized to determine the distribution of the error between cuff measurements and the corresponding arterial line values. This discrepancy was then applied as a Gaussian noise (mu=0, sigma=3.1) to the associated arterial line value, resulting in the generation of the missing cuff values.

#### Quality metrics generation

Quality metrics on patients’ records were defined and calculated to assess data quality at a segment level between two brachial cuff measurements. These metrics included the mean, variance, and extremum values for each of the derived features. The corresponding table was generated and saved locally. Abnormal segment removal was then performed using simultaneous selections for each feature, and abnormal segments were eliminated considering the following criteria:

1. 50 mmHg < segment MAP mean < 150 mmHg
2. MAP dispersion within segment < 40
3. MAP gradient within segment < 2 mmHg/s
4. MAP segment standard deviation > 1
5. MAP segment missing proportion < 20 %
6. PI missing proportion within segment < 20 %
7. PI unique values within segment > 5
8. HR missing proportion within segment < 20 %
9. PPG missing proportion within segment < 1 %

The models requiring the perfusion index feature were adapted to bypass its absence using model-specific datasets written in the Pytorch tensor format. Model-specific data loaders were defined to generate segments of input sliding windows fit for each model architecture.

### Study design: comparison to SOTA architectures

State-of-the-art architectures from the literature were compared to estimate mean arterial pressure. Depending on the models’ architecture, model features included high-frequency data but also derived features, depending on the associated models’ definition. The work presented here differentiates between calibration and calibration-free approaches. Whenever available, the code provided by the associated authors was directly used. Table 1 details the features and input window length for each model.

#### Physiologic approach based on derived features exclusively

- *Mechanistic model*: This model^9^ is a parametric model based on derived features alone. It is based on the Ohm-Poiseuille law applied to blood vessels, which states that MAP is linearly associated to HR and stroke volume (SV), such that $\widehat{MAP} = \alpha \left( \ln \left( 1 + \frac{1}{PI}\right) + \beta \right) \left( NRA + \beta '\right) HR + \delta$. It proved to achieve good results in a preliminary cohort. As one of its main features - the perfusion index - was unavailable in the VitalDB cohort, its results are reported on LaribDB exclusively. It is a parametric model that requires no training.

#### Calibration-free approaches

- *PulseNet*: This calibration-free model was excerpted from Wang et al^33^ and required no adaptation as its inputs are solely composed of raw windows of PPG and ECG sampled at 125 Hz.
- *Wave-U-Net*: Instead of predicting derived mean arterial pressure directly, the performance of the best-performing model was assessed on signal generation. The mean value per segment was generated a posteriori. This architecture was chosen for its results in signal-to-signal analysis. It is a calibration-free architecture.

#### Calibration-based approaches

- *PPG2BP-Net*: As it was presented in the introduction section, some of these models were not designed for operating room purposes and did not fit the associated criteria. To overcome this, slight adaptations were introduced to the network’s definition, notably the patient-specific SBP and DBP calibration segments for the PPG2BP-Net model^32^ were replaced. Two calibration configurations were tested: the replacement with the patient’s initial calibration measure and the last cuff measurement during the intervention. The details on this experiment are available in Supplementary Table ST1. The model architecture was reproduced and kept unchanged.
- *AnesthNet*, the proposed model, whose characteristics are detailed in the following section.

The study design is detailed in Figure 2. Model-specific dataloaders were developed to address the varying length, feature shape, and availability. For all models, a parameter search was performed using Optuna. Models were implemented using the Pytorch framework and trained on the APHP Clinical DataWarehouse infrastructure using a Nvidia GPU.

### AnesthNet model characteristics

**Figure 1:**
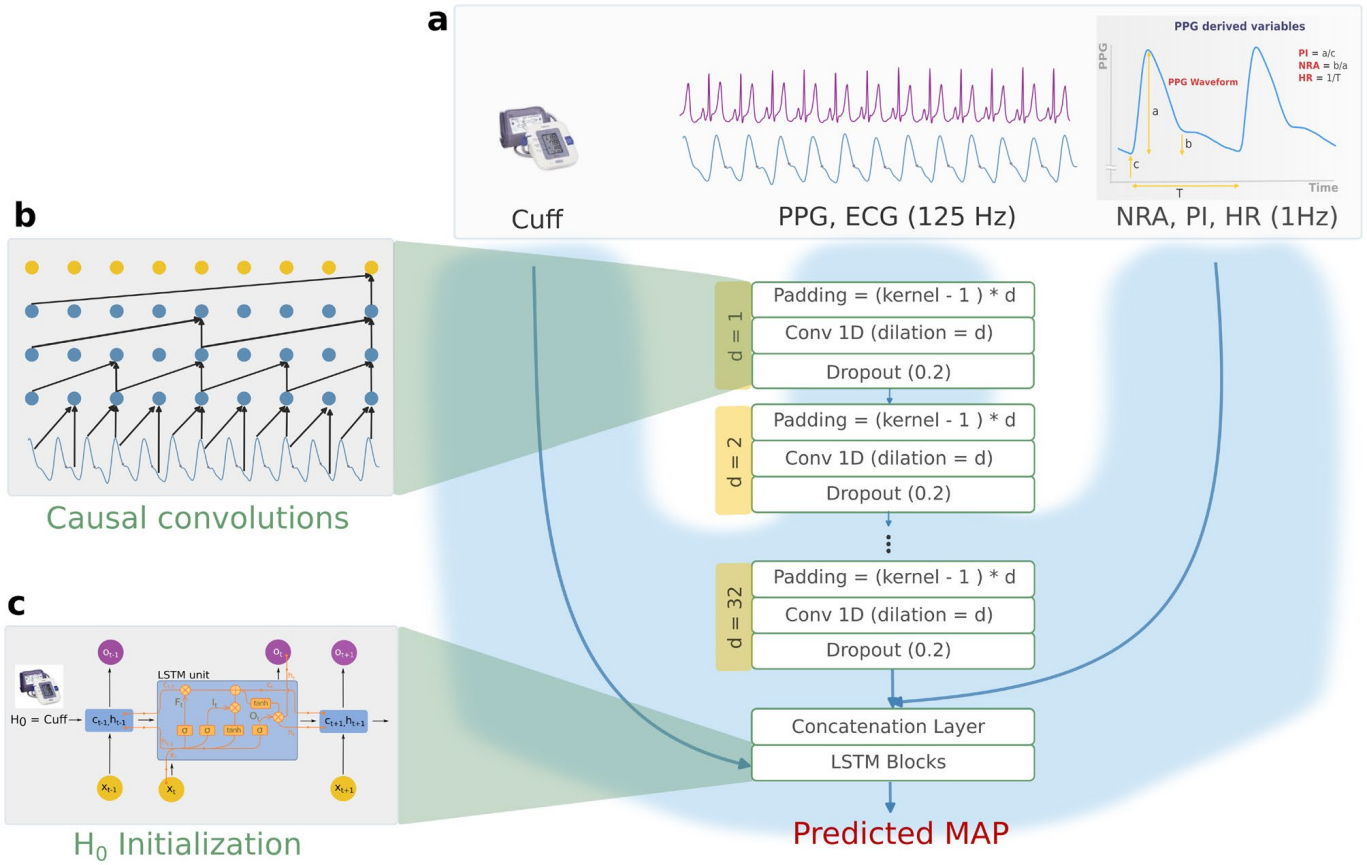
Proposed deep learning model architecture. (**a**) Multi-modal features are shaped as sequences of inputs between two cuff measurements. They include the cuff oscillometer recorded every five minutes, the high-frequency ECG and PPG (respectively acquired at 500Hz and 125Hz and downsampled to 125Hz), and PPG-derived features which carry physiological information about MAP variations: the Notch Relative Amplitude (NRA), Perfusion Index (PI), and Heart Rate (HR). (**b**) High-frequency signals are processed using a convolutional network with increased dilation to maintain causality throughout the network while increasing the receptive field within the sequence without increasing the number of parameters. The output of this network is then concatenated on the channels axis with the low-frequency derived features (NRA, PI, HR) to feed a Long Short Term Memory network. (**c**) The LSTM network is initialized using the cuff value as the initialization parameter. The output sequence corresponds to the predicted MAP sequence between the cuff measures.

To address the specificity and challenges in operating rooms, a novel deep-learning architecture was developed to estimate MAP. A major operating room characteristic is the discontinuous measure of arterial pressure using the cuff oscillometer. Thus, the objective was to interpolate the values of arterial pressure between cuff measurements. To this end, input windows were designed as segments of data between two cuff measurements.

The proposed multimodal network takes three types of input features: high-frequency waveforms from the PPG and ECG, derived cyclic features, and cuff oscillometry. Figure 1 illustrates the integration strategy for these three inputs, detailed below. As for layer choice, CNN and LSTM building blocks were used to benefit from their complementarity: the facility to optimize CNNs and the unbounded context of recurrent networks. Cuff oscillometry, which measures mean arterial pressure, was used as calibration. A CNN architecture was used to process the high-frequency waveform data; more details are available in the following paragraph regarding causal convolutions. Cyclic derived features were resampled at a 1Hz frequency and concatenated with the CNN output of the same length on the channels dimension after interpolating to preserve the time window length. More details on the ablation study that led to the use of hand-crafted features are available in Supplementary Table ST2. The resulting tensor was processed using LSTM layers the inner states of which were initialized using the segment corresponding cuff value. More details on this part of the architecture are available in the corresponding section.

#### Causal convolutions

To guarantee the feasibility of inference at runtime, and for physiological consistency, the convolutional network was built using causal convolutions, *i.e.*, using only past information to make predictions. This concept was first introduced for speech and audio generation to capture long-term dependencies^34^. Inspired by deep generative models, causal filters with dilated convolutions allowed the receptive fields to grow exponentially with depth, which is crucial to modeling the long-range temporal dependencies observed in physiological records. Because sequences were relatively long (>30,000 points), dilated convolution aimed at maximizing the memory effect on PPG waveform sequences. Dilation was increased by a factor of two for each layer. A left-side padding equal to (k - 1) * d was used where i corresponds to the layer depth within the convolutional network. Following a hyperparameter grid search, the convolutional layers’ parameters were a kernel size of 15 and a stride of 1, followed by a dropout of 0.2. Linear layers were not used as they do not preserve causality.

#### Calibration through LSTM initial inner state $h_{0}$

Recurrent layers, such as LSTM, account for the observed features’ continuity and enable us to learn across segments. Input channels from the CNN and derived features were stacked before being given as input to an LSTM network. Rather than initializing the LSTM hidden state $h_{0}$ with conventional zero or random values, we directly set $h_{0}$ to the most recent cuff-derived MAP value at the beginning of each inference segment. This approach incorporates clinically relevant calibration information into the network’s temporal processing. When new cuff measurements become available during continuous monitoring, the hidden state is reset to the updated value, enabling dynamic recalibration without parameter retraining.

#### Optimizing training to avoid I/O Bottleneck

Datasets were stored as batched arrays of windows between two cuff measurements. Models’ training time was proportional to the GPU usage proportion. Indeed, smaller data files resulted in almost immediate calculations on the GPU. To make the most of available resources, the optimal file size was assessed to improve training efficiency. The two original datasets were transformed into a common PyTorch tensor format. The file length that maximized GPU usage was one hour. This tensor format was found to minimize I/O bottlenecks while keeping storage size to a minimum. This format allowed for the design a posteriori of model-level dataloaders, adapting model processing on the fly.

**Table 1:** Model features definition and eventual calibration.

Model	High Frequency Features	Derived Features	Window length	Calibration	Operating-room adapted calibration
PulseNet	[ECG, PPG]	-	10 seconds	None	
PPG2BP-Net	[PPG]	-	10 seconds	Random SBP/DBP and PPG segment	Initial cuff and PPG segment
Wave-U-Net	[PPG, PPG’, PPG”]	-	10 seconds	None	
Mechanistic	-	[HR, DIC, PI]	5 seconds (agnostic)	Last five cuff measurements	
Proposed	[ECG, PPG]	[HR, DIC, PI]	5 minutes	Last cuff measurement	

### Statistical analysis

For each dataset, 10% of the patients were kept as a hold-out test set, while the rest of the data was split between training and validation. Results are presented on the hold-out test set of each dataset. Mean absolute error (MAE), mean error (ME), and cumulative error percentages were reported to assess model performance and the associated standard deviation to quantify error dispersion. Model predictions were all compared to the arterial line. In accordance with the AAMI standard, the Mean Error (respectively Standard Deviation of the error) should be inferior to 5 mmHg (respectively to 8 mmHg). The British Hypertension Society (BHS) defines grades of performance regarding the cumulative error percentages. The cumulative frequency of errors above the thresholds of 5, 10, and 15 mmHg was assessed for each model to compare to the standard threshold for each grade. Inference time was calculated per batch on the same machine for each dataset and then divided by the segments’ sample size. Line plots were used to represent cumulative error percentages, with colors representing each grade of the standard. Computational performance was evaluated across three processing environments: GPU, multi-core CPU, and single-core CPU configurations to simulate deployment scenarios ranging from high-performance systems to resource-constrained edge devices and microcontroller units. Single-core processing was conducted using an Intel(R) Xeon(R) CPU E5-2660 v2 @ 2.20GHz with processing restricted to a single core to emulate embedded system constraints. Additional representations such as correlation plots, Bland-Altman representations and learning curves are available in the Supplementary Figures SF1–4.

## Results

### Datasets characteristics

The study’s eligible data included 2,833 patients in VitalDB and 5,060 patients in LaribDB, for a total of 7,893 patient records. Table 2 gathers the descriptive characteristics of both datasets. For LaribDB, the dataset includes data from post-anesthetic recovery room (29%), neurosurgery (24%), surgical intensive care (19%), emergency department (7%), otolaryngology (2%), maternity (<1%) and more. In VitalDB, the main procedures reported ranged from lung and thoracic surgery (29%), digestive surgery (25%), patients from the emergency department(16 %), gynecology (5%) and urology (4%). Systolic, diastolic, and mean arterial pressure followed similar distributions with mean pressure 81.5 (± 14.3) mmHg for the training set of MAP in VitalDB (respectively 80.1 (± 20.8) mmHg in LaribDB) and 82.0 (± 14.1) mmHg in the VitalDB test set (respectively 82.4 (± 20.4) mmHg in the LaribDB test set). LaribDB patients were monitored using either a Philips (19%) or a Massimo device (81%) whereas the patients within the VitalDB database were monitored using the Tram-Rac 4A from GE Healthcare. Patients were included based on the availability of all records in the dataset, thresholds on duration, and quality metrics validation. Details about patients inclusion procedures are available in the quality metrics section and more globally in supplementary figure SF5. The study included 2,549 patients in the training set in VitalDB (respectively 4,554 in LaribDB) and 284 patients in the test set of VitalDB (respectively 506 in LaribDB). The intervals of interest were considered, namely inter-brachial periods. After segmenting the signal, a total of 41,285 non-overlapping 5-minute long windows were obtained in VitalDB (respectively 37,179 in the training set and 4,106 in the test set) and 91,663 windows in LaribDB (respectively 82,497 in the training set and 9,166 in the test set).

**Table 2:** Datasets characteristics.

Characteristics	Dataset			
	VitalDB		LaribDB	
	Train	Test	Train	Test
Number of patients, n	2549	284	4554	506
Number of 5-minute-segments, n	37179	4106	82497	9166
Age, n mean (± std)	59.1 (± 14.6)	58.1 (± 15.6)	54.1 (± 19.2)	55.3 (± 17.6)
Gender, female proportion (%)	44	45	37	38
Pressure variability, mmHg mean (± std)				
SBP	121.5 (± 22.1)	121.7 (± 21.8)	115.7 (± 28.0)	115.1 (± 28.1)
DBP	61.6 (± 13.0)	62.2 (± 12.5)	64.3 (± 20.2)	64.4 (± 18.6)
MAP	81.5 (± 14.3)	82.0 (± 14.1)	80.1 (± 20.8)	82.4 (± 20.4)
NBP (cuff)	-	-	80.6 (± 15.3)	81.2 (± 14.6)
Procedures, n (%)				
Post-anesthetic recovery room	-	-	1334	156
Neurosurgery	-	-	1116	120
Surgical intensive care	-	-	885	65
Lung/Thoracic surgery	749	83	-	-
Digestive Surgery	652	81	13	2
Emergency department	-	-	357	38
Gynecology	122	11	-	-
Otolaryngology	-	-	110	18
Urology	124	16	-	-
Maternity	-	-	65	6
Orthopedic Surgery	-	-	39	4
Other general surgery	902	93	1	2
PPG Monitoring Device, n (%)				
M1191BL SpO2 (Philips)	-	-	889	97
MightySat Rx (Massimo)	-	-	3665	409
Tram-Rac 4A (SNUADC, GE HealthCare)	2549	284	-	-

#### Considered models

**Figure 2:**
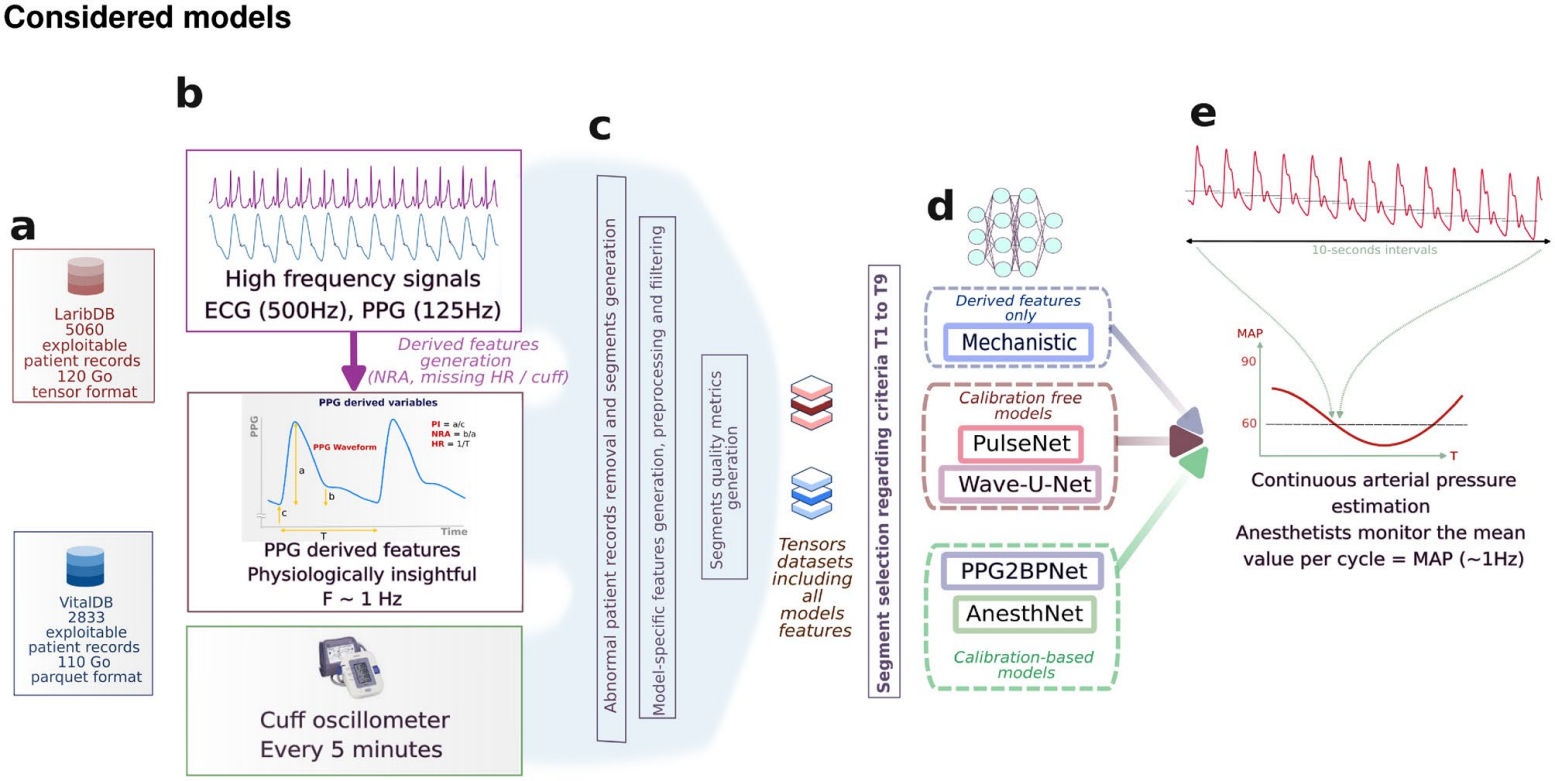
Study design. (**a**) Exploitable datasets sizes: LaribDB (5060 patients) and VitalDB (2833 patients). (**b**) Multimodal features categories which include high-frequency data (ECG, PPG), PPG-derived features, and the cuff oscillometer. (**c**) Preprocessing steps, filtering and ready-to-train datasets generation. (**d**) State-of-the-art models compared in this study, including physiology-based, calibration-free and calibration-based approaches. (**e**) Each model takes as input a combination of non invasive features and produces a prediction of MAP per window of data. Results are then aggregated per patient to generate the main results metrics.

This comparative study assessed mechanistic, calibration-based, and calibration-free state-of-the-art models. When relevant, architectures were adapted to operating-room settings, notably regarding realistic calibration. Random systolic and diastolic segments were systematically replaced by the last cuff measurement. The most straightforward calibration-free approach was composed of a ResNet architecture using only the raw ECG and PPG^33^. A slightly more complex calibration-free architecture was based on the U-Net architecture originally designed for audio signals, with successive downsampling and upsampling blocks to reconstruct the desired time series^34^. This model was applied to raw ECG and PPG signals as well. Then, calibration-based models included notably PPG2BP-Net, a CNN-MLP network calibrating using a pair of random PPG and arterial pressure segments for each entry. A multilinear model inspired by physiology was trained on low-frequency features derived from the raw signal of the PPG and recalibrated every five minutes using the cuff. In the following section, this model will be referred to as the mechanistic model.

The model proposed, AnesthNet, estimates arterial pressure during the interval between cuff measurements, which guided the design of the study towards its input characteristics as segments of five consecutive minutes of data. Input features belong to three categories: cuff calibration, high-frequency PPG and ECG signals, and low-frequency features that are physiologically informative. High-frequency features were fed to a causal convolutional network to account for long-term dependencies within segments, and later on concatenated with low-frequency inputs into LSTM recurrent layers. Calibration is performed using the LSTM inner state affected with the cuff value whenever available.

### Models performance assessment

Performance metrics were chosen based on international standards criteria. According to the AAMI standard, the Mean Error (respectively Standard Deviation of the error) should be inferior to 5 mmHg (respectively to 8 mmHg). Cumulative error percentages for 5, 10, and 15 mmHg were assessed for all models to compare their performance to the British Hypertension Society (BHS) standard criteria. As a result, mean absolute error (MAE) and mean error (ME) were reported along with their associated standard deviation, as much as cumulative error percentages to compare model performance.

Table 3 gathers the results of each model on VitalDB and LaribDB. The AnesthNet model reached an MAE of 4.6 ($\pm 4.6$) mmHg on VitalDB and 3.8 ($\pm 5.7$) mmHg on LaribDB. The PulseNet reached an MAE of 11.6 ($\pm 9.3$) mmHg on VitalDB and 11.7 ($\pm 11.1$) mmHg on LaribDB and PPG2BP-Net reached an MAE of 5.3 ($\pm 4.7$) mmHg on VitalDB and 8.1 ($\pm 7.2$) mmHg on LaribDB. The Wave-U-Net model reached a performance of 10.2 ($\pm 8.7$) mmHg on VitalDB and 11.5 ($\pm 9.2$) mmHg on LaribDB. As for cumulative error percentages, the AnesthNet model achieved a grade A performance on both databases. PPG2BP-Net achieved a grade B performance on VitalDB and grade C on LaribDB. The mechanistic model reached a grade C performance on LaribDB. Other models did not meet the BHS standard criteria for the lowest grade, grade C. These performances can be compared to previously published results obtained for BP estimation in Supplementary Table ST3.

**Table 3:** Models performance comparison among various learning- and PPG-based cuffless BP estimation architectures based on the Mean Error and Mean Absolute Error metrics and BHS standard metrics. Estimation was based on i) AAMI standard: The number of test subjects needs to be higher than 85, the ME should be $\le$ ±5 mmHg, and the SD of error should be $\le$ 8 mmHg; and ii) BHS standard: The grades based on the BHS standard are given based on the error percentage as follows: if 60%, 50%, and 40% of errors $\le$ 5 mmHg, then grades A, B, and C are given, respectively; if 85%, 75%, and 65% of errors $\le$ 10 mmHg, then grades A, B, and C, are given, respectively; if 95%, 90%, and 85% of errors $\le$ 15 mmHg, then grades A, B, and C, are given, respectively. The highlights in bold represent the model reaching the best performance.

Database	Models	Error Metrics		BHS Error Metrics			
		ME (mmHg)	MAE (mmHg)	e $\le$ 5 mmHg (%)	e $\le$ 10 mmHg (%)	e $\le$ 15 mmHg (%)	BHS Grade
VitalDB	Mechanistic	–	–	–	–	–	–
	PulseNet	-1.1 (± 14.8)	11.6 (± 9.3)	33.1	60.1	78.7	Below C
	Wave-U-Net	-2.8 (± 12.8)	10.2 (± 8.7)	30.2	57.0	76.5	Below C
	PPG2BP-Net	0.67 (± 6.7)	5.5 (± 5.1)	57.7	83.5	93.4	Grade B
	AnesthNet (Proposed)	**0.3 (**± **6.3)**	**4.6 (**± **4.7)**	**65.7**	**88.3**	**96.1**	Grade A
LaribDB	Mechanistic	-0.8 (± 14.2)	7.9 (± 11.9)	60.1	77.2	84.1	Grade C
	PulseNet	3.7 (± 15.4)	11.7 (± 11.1)	31.8	59.2	79.4	Below C
	Wave-U-Net	-3.6 (± 14.3)	11.5 (± 9.2)	29.4	55.1	73.9	Below C
	PPG2BP-Net	-2.42 (± 10.4)	8.1 (± 7.2)	40.8	70.1	86.3	Grade C
	AnesthNet (Proposed)	**0.2 (**± **6.8)**	**3.8 (**± **5.7)**	**72.1**	**91.2**	**95.3**	Grade A

**Table 4:** Models performance regarding mean inference time. Mean inference time was defined as the time taken by each model to perform inference on a batch of features. It was averaged over 15 batches and latencies were reported in milliseconds.

Database	Models	Inference Time	
		GPU (ms)	24-core CPU (ms)
VitalDB	Mechanistic	–	–
	PulseNet	2.3	9.1
	Wave-U-Net	2.6	11.6
	PPG2BP-Net	**1.8**	**3.7**
	AnesthNet (Proposed)	3.1	21
LaribDB	Mechanistic	-	1.8
	PulseNet	**1.5**	6.4
	Wave-U-Net	2.6	27.4
	PPG2BP-Net	1.6	**3.6**
	AnesthNet (Proposed)	3.3	24

#### Performance regarding models latency

Table 4 gathers the results regarding models latency comparison. For all models, the mean latency remained within the same order of magnitude for GPU and 24 CPU cores, ie, below 30 milliseconds (ms) per batch. The lowest mean latencies were observed for PPG2BP-Net and PulseNet for a respective mean latency per batch of 1.8 ms on VitalDB and 1.6 ms on LaribDB. The use of a GPU showed an overall improvement by a factor of 2 to 10 compared to the usage of 24 CPU cores. For the proposed AnesthNet model, the mean latency introduced by the inference process was respectively 3.1 and 3.3 milliseconds for VitalDB and LaribDB. Supplementary Table ST4 shows that even under the deliberately constrained conditions of single-core CPU execution, the latency of all models remained below 500 milliseconds. These latency values are to be compared to the intrinsic latency of five seconds introduced by the operating room acquisition unit at data collection time.

### The impact of distance to cuff calibration

To address the variability of practices or conventions observed between medical sites, the models’ robustness to the variation of brachial cuff frequency was assessed. The associated datasets were generated for cuff frequencies of 3, 5, and 10 minutes and the associated windows of data. Results were compared for calibrated models only and are presented in Table 5. On VitalDB, PPG2BP-Net reached a MAE of 4.8 (± 4.6) mmHg for three-minute-intervals (respectively 5.5 (± 5.1) mmHg for five-minute-intervals and 7.3 (± 5.9) mmHg for ten-minute-intervals). On the same database, the proposed AnesthNet model reached a MAE of 3.9 (± 4.1) mmHg for three-minute-intervals (respectively 4.6 (± 4.7) mmHg for five-minute-intervals and (6.1 ± 5.8) mmHg for ten-minute-intervals). On LaribDB, PPG2BP-Net reached a MAE of 7.8 (± 6.9) mmHg for three-minute-intervals (respectively 8.1 (± 7.2) mmHg for five-minute-intervals and 8.9 (± 7.8) mmHg for ten-minute-intervals). On the same database, the AnesthNet model reached a MAE of 3.3 (± 5.1) mmHg for three-minute-intervals (respectively 3.8 (± 5.7) mmHg for five-minute-intervals and 5.7 (± 6.6) mmHg for ten-minute-intervals). AnesthNet proved more robust to an increased duration between two brachial cuff measurements and obtained significantly better results, notably on the more challenging dataset LaribDB.

**Table 5:** Calibration-based models performance regarding distance to cuff calibration. Segments of respectively 3, 5 and 10 minutes were generated to model the variation of cuff measurement frequency. Inference is performed during these intervals for calibrated models.

Variable	Metric (Unit)	VitalDB		LaribDB	
		PPG2BP-Net	AnesthNet	PPG2BP-Net	AnesthNet
Cuff - 3 minutes	ME (mmHg)	-0.76 (± 6.1)	0.1 (± 4.85)	0.7 (± 10.4)	0.8 (± 6.1)
	MAE (mmHg)	4.8 (± 4.6)	**3.9 (**± **4.1)**	7.8 (± 6.9)	**3.3 (**± **5.1)**
	e $\le$ 5 mmHg (%)	60.9	71.1	41.9	81.9
	e $\le$ 10 mmHg (%)	86.0	90.5	71.2	93.9
	e $\le$ 15 mmHg (%)	94.9	96.5	87.1	97.1
Cuff - 5 minutes	ME (mmHg)	0.67 (± 6.7)	0.3 (± 6.3)	-2.42 (± 10.9)	0.2 (± 6.8)
	MAE (mmHg)	5.5 (± 5.1)	**4.6 (**± **4.7)**	8.1 (± 7.2)	**3.8 (**± **5.7)**
	e $\le$ 5 mmHg (%)	57.7	65.7	40.8	72.1
	e $\le$ 10 mmHg (%)	83.5	88.3	70.1	91.2
	e $\le$ 15 mmHg (%)	93.4	96.1	86.3	95.3
Cuff - 10 minutes	ME (mmHg)	-5.0 (± 8.1)	0.9 (± 7.8)	-0.4 (± 11.8)	0.5 (± 8.7)
	MAE (mmHg)	7.3 (± 5.9)	**6.1 (**± **5.8)**	8.9 (± 7.8)	**5.7 (**± **6.6)**
	e $\le$ 5 mmHg (%)	42.2	55.2	37.8	62.1
	e $\le$ 10 mmHg (%)	73.8	79.8	66.1	83.7
	e $\le$ 15 mmHg (%)	89.1	91.9	82.9	92.1

## Discussion

This multicentric study offers valuable insights into the performance of state-of-the-art models for non-invasive blood pressure estimation in the operating room, using the largest datasets available to date. Both recent-and-competitive and traditional-yet-effective approaches were included to reflect the range of existing methods. Comparing and evaluating these models from an anesthetist’s perspective emphasizes deployable architectures suitable for practical use and underscores the performance disparity between cuff-based and cuffless methods. The proposed architecture, AnesthNet, is a multimodal network that takes both raw waveform and cyclic features, along with brachial cuff measurements, and proposes estimates between cuff measurements using built-in deep learning model parameters. Generally speaking, calibration-based architectures performed better than calibration-free and mechanistic approaches. More specifically, AnesthNet outperformed the other models independently of the interval between cuff measurements. Notably, LaribDB appears to be more discriminative across models, revealing larger performance differences, especially with PPG2BP-Net, which did not meet the BHS grade C. This may be due to the greater variability and complexity in signal collected in the LaribDB dataset compared to benchmark datasets like VitalDB. The integration of a clinical prior via LSTM initialization in the AnesthNet could have contributed to its robustness and generalizability across possibly more challenging, real-world conditions.

In practice, calibration is always available, but the frequency with which it is taken may vary depending on the stage of the operation or the usual practices in a hospital. Resilience to varying calibration is crucial to ensure the robustness of an estimator within real-life conditions. Although providing the best results, the calibration-based models were sensitive to the distance to the last cuff. However, performance error remained acceptable for the usual frequency ranges of cuff measurement. The use of the proposed MAP estimation is compatible with the conventional use of cuff calibration. As demonstrated in Table 5, calibration-based models’ performance degrades progressively as the calibration interval increases, with none of the models achieving an A quality level beyond 10 minutes from calibration. This temporal degradation pattern represents a limitation in blood pressure estimation, indicating that recalibration through additional oscillometric measurements becomes necessary beyond this threshold. The observed performance decline underscores the complementary nature of technical approaches with clinical practice, where the system serves as an assistant to clinicians by providing real-time estimation while signaling when recalibration is required to maintain accuracy.

All models provided acceptable results regarding latency. Using a GPU systematically and significantly reduced inference time. The AnesthNet approach, along with the Wave-U-Net architectures, showed the highest benefit of GPU usage, which is consistent with their larger number of parameters. Nonetheless, all models’ prediction times respected the order of magnitude of current measurement methods ($\Delta t$ < 1 second per sample) and are compatible with operating room inference infrastructure. While the concept of a trade-off between performance and inference time could be discussed, the notable differences in model performance suggest that the accuracy of predictions prevails here.

Regarding the interactions between features, simultaneous and synchronous monitoring of ECG and PPG provided better results. Physiologically, this could be explained by the fact that it enables the estimation of the pulse arrival time (PAT), defined as the time interval between the R-wave of the ECG and the arrival of the corresponding pulse wave at a peripheral site, typically measured using a PPG sensor. As a surrogate of the pulse transit time (PTT), and often used as an indicator of cardiovascular function, the PAT is related to arterial stiffness and blood pressure, notably in high frequency ranges^35^. However, since the PAT varies by the same orders of magnitude as the shifts that can be observed in signals due to measurement artefacts, its systematic calculation remains untrustworthy.

Traditional research in this domain evaluates performance outcomes using internationally recognized benchmarks such as the AAMI and BHS standards, predicated on estimating near real-time beat-to-beat arterial pressure values. These could be over-optimistic in the case of an over-representation of patients with a low BP variation range. Apart from this, modelling the occurrence of hypotension is also a subject in itself. Some approaches to framing the problem also allow for a more specific visualization of performance concerning hypotension, such as classifying hypotensive event detection; however, this relies on a rather unsettled clinical definition of IOH. Also, such an approach does not address the issue of prediction uncertainty, though it could provide more representative conclusions regarding model accuracy. Moreover, considering the growing complexity of the models used, it could foster clinical acceptability. Future work will notably attempt to assess the model uncertainty using state-of-the-art methods, such as the conformal uncertainty prediction framework^36^. Such an approach would make it possible to add to the arterial pressure estimator a quantification of the risk associated with the uncertainty of the predictions. The deployment of the model in the operation room will also be performed to test the system in real-life conditions. In future investigations, examining systolic blood pressure (SBP) and diastolic blood pressure (DBP) as distinct variables could also provide valuable insights beyond those captured by mean arterial pressure alone. While MAP is central to anesthetists, analyzing SBP and DBP separately may reveal differential associations with clinical outcomes, as these parameters reflect distinct aspects of cardiac function and vascular dynamics. Such an approach could enhance our understanding of the independent contributions of systolic and diastolic pressures to advanced methods in monitoring or cardiology.

### Conclusion

In this study, various architectures were compared for the non-invasive estimation of Mean Arterial Pressure (MAP) using operating room data. Our proposed model combines causal convolutions on physiological signals to capture long-term dependencies with an initialization strategy for deep learning parameters. Specifically, the inner state of Long Short-Term Memory (LSTM) networks was initialized using field calibration data from cuff oscillometry, leveraging domain knowledge and enabling real-time capabilities. Our model demonstrated superior performance compared to existing architectures on both databases, while maintaining inference times compatible with near real-time deployment.

## Supplementary Information

Supplementary Information.

## Data Availability

VitalDB dataset: https://vitaldb.net/dataset/ The acquisition and release of the data was approved by the Institutional Re- view Board of Seoul National University Hospital (H-1408-101-605). The study was also registered at clinicaltrials.gov (NCT02914444). LaribDB dataset: The data will be made available from authors upon reasonable requests. The readers can contact Jona Joaquim for more information.
