# Supplementary material for: Harnessing operating room signals to estimate mean arterial pressure with AnesthNet
Source: Sci Rep. 2025 Sep 30;15:33988. doi: 10.1038/s41598-025-12341-8 (PMC12484649; doi:10.1038/s41598-025-12341-8)
Supplement: Supplementary file 1 — Supplementary Information. [file 41598_2025_12341_MOESM1_ESM.pdf]

Supplementary Information

Database	Models	Error Metrics		BHS Error Metrics		
		ME (mmHg)	MAE (mmHg)	e ≤ 5 mmHg	e ≤ 10 mmHg	e ≤ 15 mmHg
VitalDB	PPG2BP-Net: Initial cuff	1.3 (± 9.1)	6.9 (± 6.2)	48.2	77.3	90.6
	PPG2BP-Net: Last cuff	0.67 (± 6.7)	5.5 (± 5.1)	57.7	83.5	93.4
LaribDB	PPG2BP-Net: Initial cuff	6.9 (± 12.8)	10.7 (± 10.1)	35.6	59.7	74.3
	PPG2BP-Net: Last cuff	-2.42 (± 10.4)	8.1 (± 7.2)	40.8	70.1	86.3

Supplementary table ST1: PPG2BP-Net performance comparison depending on the replaced calibration definition. A conservative approach close to the calibration performed by the authors is to calibrate using a unique calibration point corresponding to the beginning of the intervention. A more precise approach leveraging the latest measure from the operating room is to use the previous cuff measure. In the main results, this model's results are presented with the configuration that maximized performance, that is to say, the latest.

Dataset	Architecture	ME (mmHg)	MAE (mmHg)
VitalDB	Dilated CNN	-1.1 (± 6.7)	4.8 (± 5.1)
	Dilated CNN + Hand-crafted features	0.3 (± 6.3)**	4.6 (± 4.7)**
LaribDB	Dilated CNN	-0.4 (± 7.2)	3.9 (± 6.0)
	Dilated CNN + Hand-crafted features	0.2 (± 6.8)**	3.8 (± 5.7)**

Supplementary Table ST2: Ablation study regarding hand-crafted features. The ablation study was conducted on VitalDB. For each dataset, a paired t-test revealed that the error distributions of our model and the other model were significantly different ($p<0.001$), as indicated by the double asterisk in the table.

Reference	Model name	Dataset (dataset category)	Number of training subjects	Number of test subjects	SBP error (mmHg)	DBP error (mmHg)	Obtained MBP error (mmHg)
[18]	Adaboost	MIMIC II (ICU)	At least 441	At least 41	-0.05 (± 8.9)	0.18 (±4.17)	-
[18]	Random Forest Regressor	MIMIC II (ICU)	At least 441	At least 41	0.15 (± 10.6)	0.19 (± 4.73)	-
[18]	SVR	MIMIC II (ICU)	At least 441	At least 41	-0.9 (± 16.71)	-0.6 (±7.5)	-
[21]	ABP-Net	ICU DB (ICU)	1620	162	1.64 (±7.42)	-0.28 (±5.81)	-2.8 (± 12.8)
[25]	U-Net	UCI Dataset (ICU)	unknown	unknown	0.06 (± 20.9)	-2.46 (± 10.8)	-
[32]	PPG2BPNet	VitalDB (Anesthesia)	2987	410/797 (whole: hold-out)	-0.231 (±10.263)	0.062 (±6.252)	0.67 (± 6.7)
[33]	PulseNet	VitalDB / MIMIC IIII (Anesthesia/ ICU)	2506	279	0.8 (± 18.6)	0.5 (± 12.3)	-1.1 (± 14.8)

Supplementary Table ST3: Performance Comparison of Blood Pressure Estimation Models. This table shows the performance obtained by other models on previously published work. The ‘dataset category‘ column stands for anesthesia versus intensive care unit, to account for the patient admission category. The obtained MBP error corresponds to the model performance reached in our study for the VitalDB dataset.

Model name	Number of parameters	Latency on single-core CPU (ms)
Mechanistic	5	86
PulseNet	3,844,865	266
Wave-U-Net	5,484,695	415
PPG2BP-Net	1,136,763	154
AnesthNet	2,978,873	197

Supplementary Table ST4: Resource constrained evaluation. We tested all models using single-core CPU execution. This represents a severely limited processing environment relative to the multi-core systems standard in modern operating rooms. Under these restrictive conditions, all models achieved latencies below 500 milliseconds. We note that these performance benchmarks were deliberately conservative to establish computational feasibility even in resource-constrained settings. These processing times are substantially faster than the 5-second acquisition delay inherent to the operating room data collection system, confirming that model inference does not introduce computational bottlenecks into the clinical pipeline.

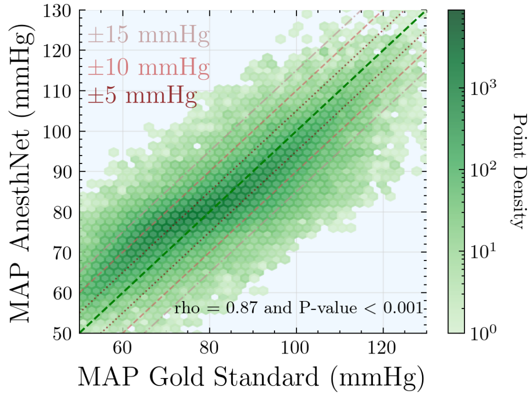

(a) AnesthNet train set correlation plot

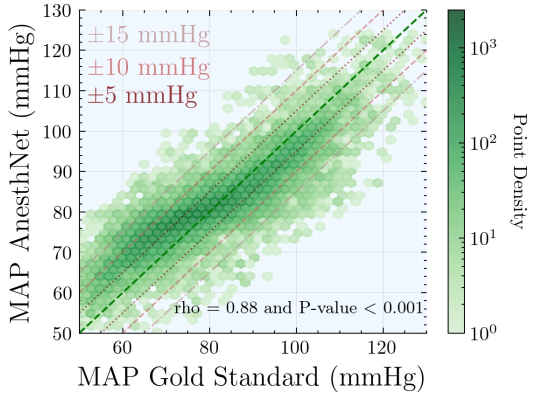

(b) AnesthNet test set correlation plot

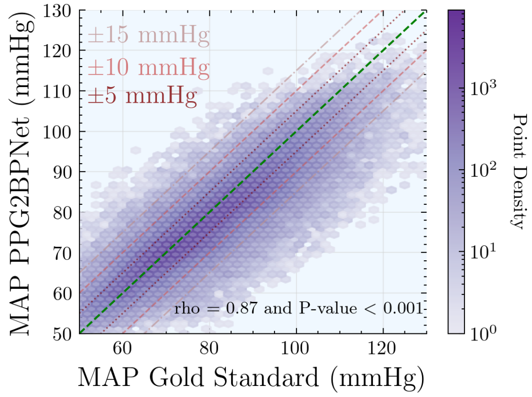

(c) PPG2BPNet train set correlation plot

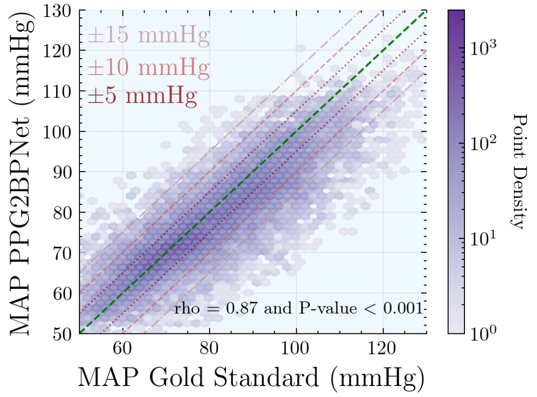

(d) PPG2BPNet test set correlation plot

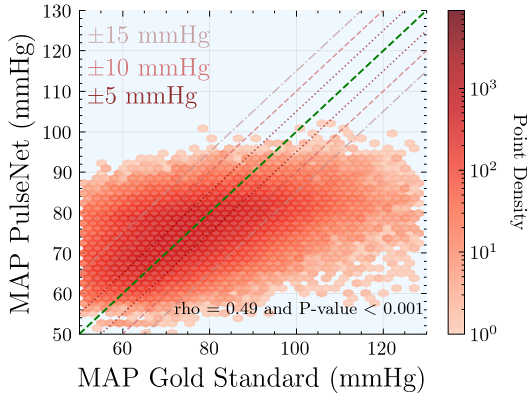

(e) PulseNet train set correlation plot

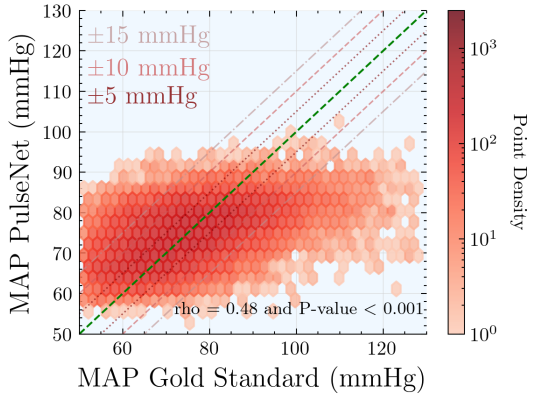

(f) PulseNet test set correlation plot

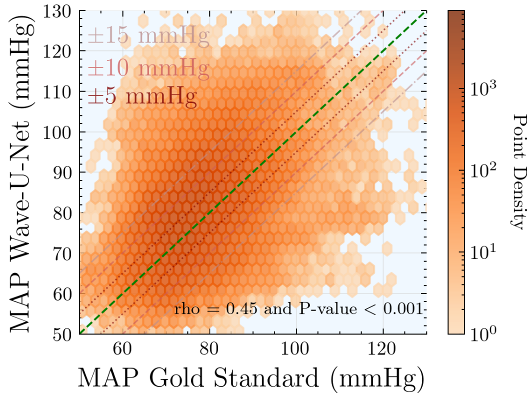

(g) Wave-U-Net train set correlation plot

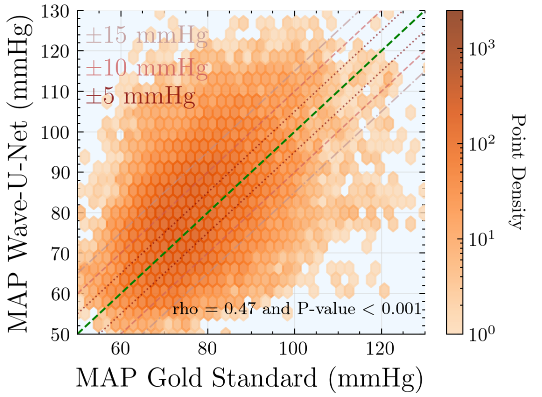

(h) Wave-U-Net test set correlation plot

Supplementary Figure SF1: Models predictions correlation plots against catheter gold standard on VitalDB Dataset. Considering the high dataset size, hexagonal binning was used to represents groups of points with a gridsize of 25. Logarithmic scale is used to display point density.

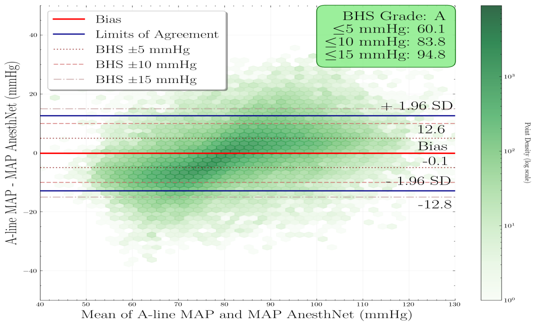

(a) AnesthNet train set correlation plot

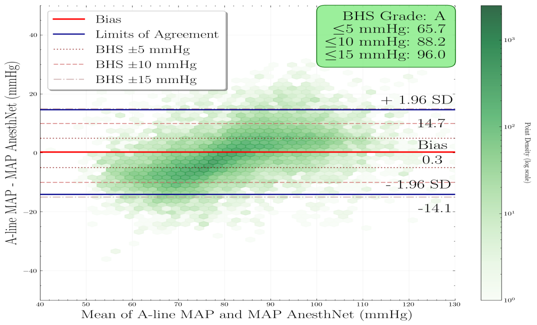

(b) AnesthNet test set correlation plot

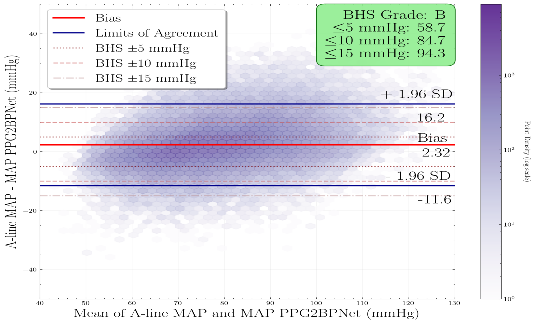

(c) PPG2BPNet train set correlation plot

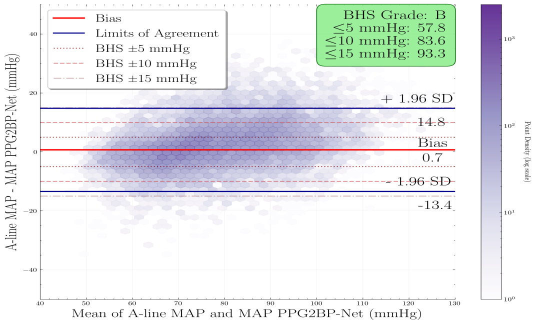

(d) PPG2BPNet test set correlation plot

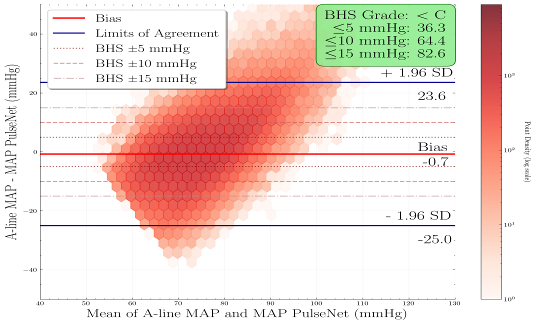

(e) PulseNet train set correlation plot

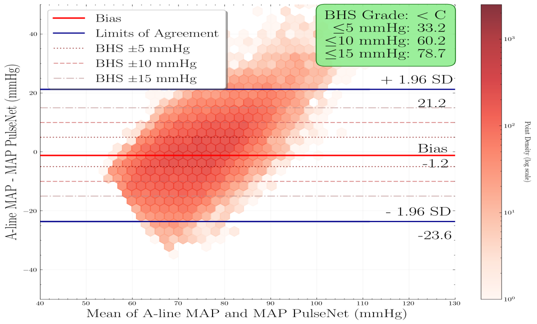

(f) PulseNet test set correlation plot

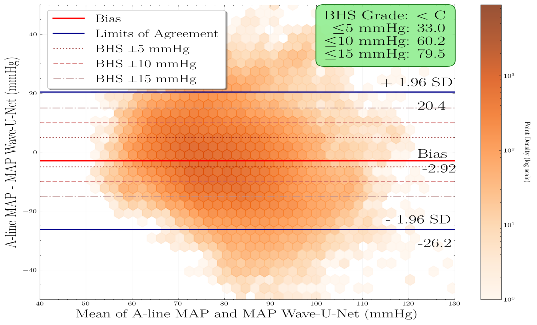

(g) Wave-U-Net train set correlation plot

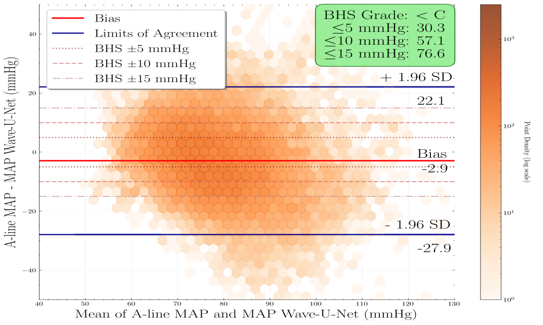

(h) Wave-U-Net test set correlation plot

Supplementary Figure SF2: Models predictions Bland-Altman representations against catheter gold standard on VitalDB Dataset. Considering the high dataset size, hexagonal binning was used to represents groups of points with a gridsize of 25. Logarithmic scale is used to display point density.

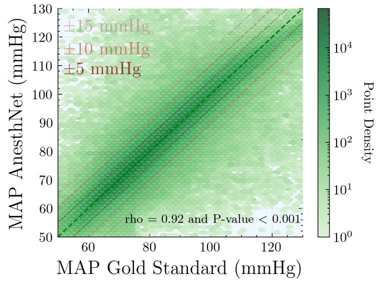

(a) AnesthNet correlation plot on LaribDB test set

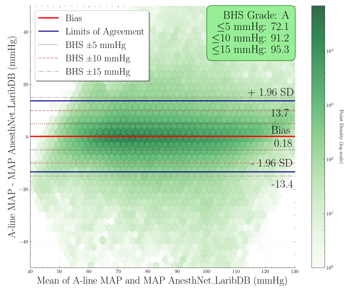

(b) AnesthNet Bland-Altman on LaribDB test set

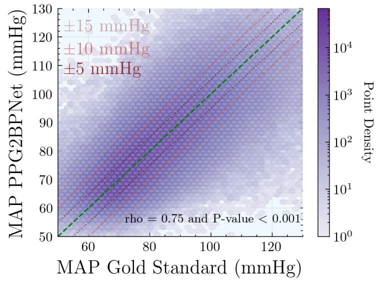

(c) PPG2BP-Net correlation plot on LaribDB test set

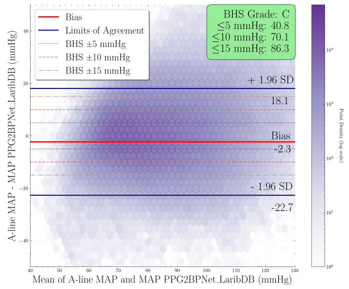

(d) PPG2BPNet Bland-Altman on LaribDB test set

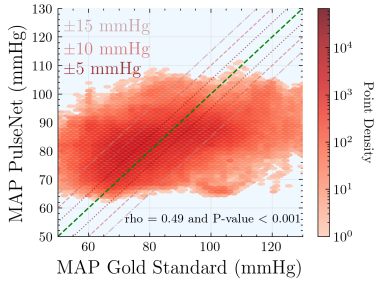

(e) PulseNet correlation plot on LaribDB test set

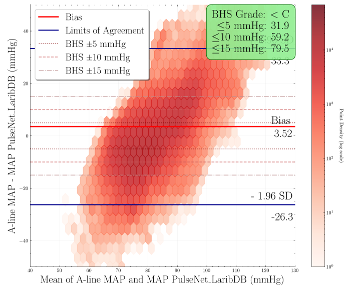

(f) PulseNet Bland-Altman plot on LaribDB test set

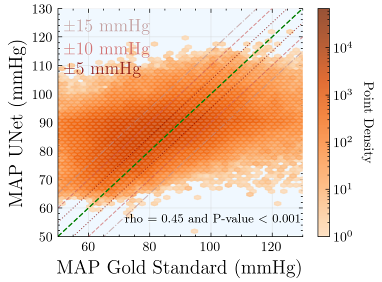

(g) Wave-U-Net correlation plot on LaribDB test set

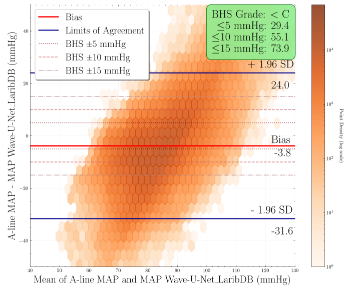

(h) Wave-U-Net Bland-Altman plot on LaribDB test set

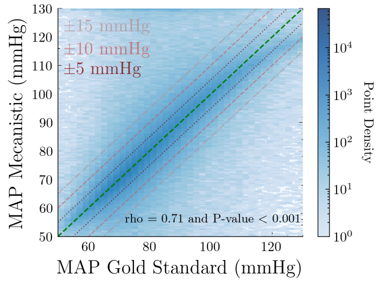

(i) Mechanistic correlation plot on LaribDB test set

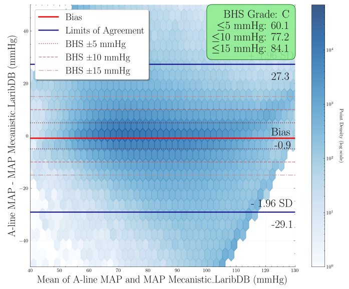

(j) Mechanistic Bland-Altman plot on LaribDB test set

Supplementary Figure SF3: Models' predictions correlation and Bland-Altman plots against arterial line gold standard on LaribDB Dataset test set. Considering the high dataset size, hexagonal binning was used to represents groups of points with a gridsize of 25. Logarithmic scale is used to display point density.

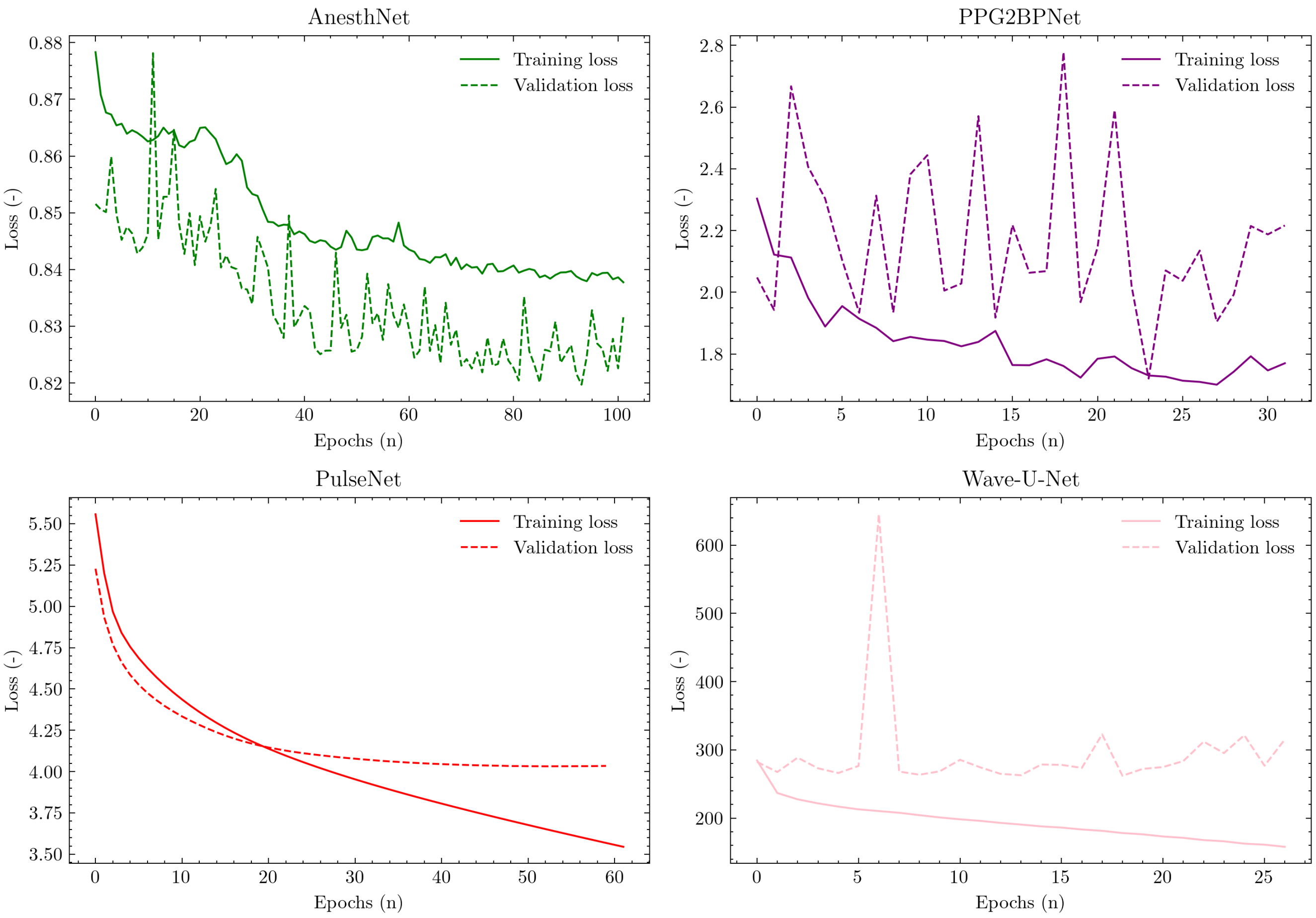

Supplementary Figure SF4: Models training and validation curves. Considering training trends, PulseNet training and validation appear very smooth compared to the others, which can be explained by the multiple regularization techniques chosen by the authors. Calibration-free models tended to converge earlier, with Wave-U-Net diverging from the first epochs. On the other hand, the proposed model is slower to converge and the validation loss decreases until 100 epochs, as opposed to 18 pour PulseNet. The validation accuracy of PPG2BP-Net appears to be unstable, although the training accuracy does converge after 25-28 epochs.

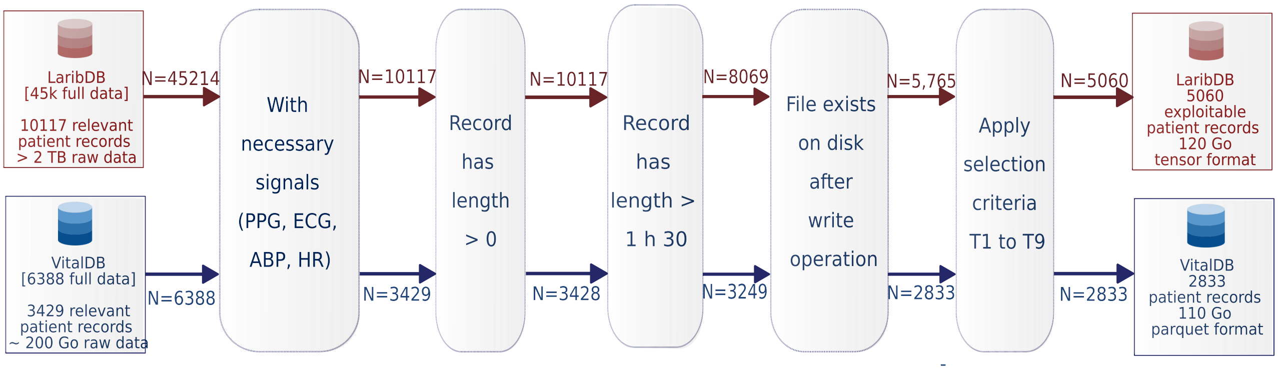

Supplementary Figure SF5: Detailed patient selection process and criteria for both databases.
